# Effect of cytoplasmic fragmentation on embryo development, quality, and pregnancy outcome: a systematic review of the literature

**DOI:** 10.1186/s12958-024-01217-7

**Published:** 2024-05-14

**Authors:** Ariella Yazdani, Iman Halvaei, Catherine Boniface, Navid Esfandiari

**Affiliations:** 1 Department of Obstetrics, Gynecology, and Reproductive Sciences, University of Vermont Medical Center, The Robert Larner College of Medicine at the University of Vermont, Burlington, VT 05405 USA; 2 Department of Anatomical Sciences, Faculty of Medical Sciences, Tarbiat Modares University, Tehran, Iran; 3 Present address: Obstetrics and Gynecology Institute, Cleveland Clinic Foundation, Cleveland, OH 44195 USA; 4 Division of Reproductive Endocrinology and Infertility, Department of Obstetrics and Gynecology and Reproductive Sciences, University of Vermont, 111 Colchester Avenue, Burlington, Vermont 05401 USA

**Keywords:** Fragmentation, Embryo development, Implantation, In vitro fertilization, Pregnancy outcome

## Abstract

The role of cytoplasmic fragmentation in human embryo development and reproductive potential is widely recognized, albeit without standard definition nor agreed upon implication. While fragmentation is best understood to be a natural process across species, the origin of fragmentation remains incompletely understood and likely multifactorial. Several factors including embryo culture condition, gamete quality, aneuploidy, and abnormal cytokinesis seem to have important role in the etiology of cytoplasmic fragmentation. Fragmentation reduces the volume of cytoplasm and depletes embryo of essential organelles and regulatory proteins, compromising the developmental potential of the embryo. While it has been shown that degree of fragmentation and embryo implantation potential are inversely proportional, the degree, pattern, and distribution of fragmentation as it relates to pregnancy outcome is debated in the literature. This review highlights some of the challenges in analysis of fragmentation, while revealing trends in our evolving knowledge of how fragmentation may relate to functional development of the human embryos, implantation, and pregnancy outcome.

## Introduction

Human preimplantation embryo scoring systems have been widely used to predict blastocyst development and implantation rate after in-vitro fertilization (IVF). The grading of embryos on day-2 and -3 after fertilization is largely subjective and interpretation varies across IVF laboratories, as it is commonly based on morphological appearance. Characteristics in early embryo grading schema include the amount of cytoplasmic fragmentation (CF) during early cleavage, speed of cellular division, number, size, and symmetry of cells (blastomeres). As defined by the Istanbul consensus workshop on embryo assessment, a fragment is “an extracellular membrane-bound cytoplasmic structure that is < 45 µm diameter in a day-2 embryo and < 40 µm diameter in a day-3 embryo” [1]. There are several different systems to evaluate embryo morphology including Hill’s scoring system [2] Cummins' grading system [3] ASEBIR grading system [1], the UK/ACE grading scheme [4]; each system has its own classification for degree of fragmentation as well as embryo grade. This heterogeneity further complicates analysis of fragmentation in relation to outcomes.

CF has been shown to occur early in embryonic division and is a common phenomenon seen in embryos cultured in vitro. CF has traditionally been used as a metric of embryo implantation potential [3, 5–7]. The amount and pattern of fragments are analyzed in early development, incorporated into the embryo grade depending on grading system, and used to help select the most developmentally competent embryo to be transferred during an IVF cycle. This classification system is important as a proportion of embryos within a single cohort will not successfully develop to the blastocyst stage in vitro. Although there are various contributing factors to an embryo’s developmental capacity and viability, it is largely agreed upon that fragmentation plays an important role. It seems that the etiology of embryo fragmentation is not fully understood but it may be related to several factors like gamete quality, culture condition, and genetic abnormalities in the embryo [8]. It is difficult to directly compare and quantify relative degrees of fragmentation across studies. However, it has been repeatedly shown that the extent of fragmentation and implantation potential are inversely proportional [5, 7, 9–21]. While a low degree of fragmentation does not seem to significantly impact embryo viability, severe fragmentation does [7, 22, 23]. Alongside the cell to cytoplasmic ratio, the pattern and distribution of fragmentation influence the developmental quality of the embryo [7, 24]. There are two main patterns of embryo cytoplasmic fragments: scattered and concentrated. The former is characterized by fragment contact within several blastomeres and is related to aneuploidy [25]. Time-lapse studies have shown that fragmentation is thought to be a dynamic process, where some fragments can be expelled or reintroduced into the cells as the embryo continues to divide [25, 26]. Fragments can also easily move or rotate around the associated blastomere and change their position in the embryo [27].

Current grading systems used to evaluate cleavage-stage embryos are largely based on day-2 or -3 morphology. This can be problematic, as developmental growth of an embryo is variable and the grade of a developing embryo at one point in time is not guaranteed to persist. For example, studies have suggested that embryo selection on day-2 or -3 based on morphological grade can be unreliable and lead to negative pregnancy outcomes [28–30]. Accordingly, new parameters for predicting implantation success have been proposed including extended embryo culture to the blastocyst stage to day-5, -6 or -7 [31]. Delaying embryo transfer to the blastocyst stage is advantageous as it can limit the number of unsuccessful embryo transfers and biochemical pregnancies or clinical pregnancy losses in IVF. While there are multiple reports on the impact of cleavage-stage embryo quality on blastocyst formation and blastocyst quality [32, 33], few have specifically looked at the degree of fragmentation as a predictive variable.

In this systematic review, we comprehensively reviewed the available literature on the origin and characteristics of CF, factors affecting CF, and the effect of CF and fragment removal on embryo development and pregnancy rate.

## Materials and methods

A search was conducted on October 10, 2023, using PubMed and Google Scholar databases in accordance with Preferred Reporting Items for Systematic Reviews and Meta-Analysis guidelines [34]. In PubMed, the search terms “embryo*[tw] OR cleavage stage [tw] OR "Embryonic Structures"[Mesh] OR "Embryonic Development"[Mesh] OR "Embryo, Mammalian"[Mesh] OR "Cleavage Stage, Ovum"[Mesh]” AND “cytoplasm*[tw] AND fragment*[tw] AND “(Blastocyst*[tw] OR "Blastocyst"[Mesh]) AND (form* OR develop* OR quality*)” were used. A title search in Google Scholar using search terms as above and “embryo cytoplasm fragmentation”, “blastocyst quality”, “blastocyst development” was performed. Only full-text publications in English were included. Full-text articles which did not have any mention of cytoplasmic or embryo fragmentation were excluded, however articles which mentioned both DNA fragmentation and CF were included. Since most of the studies discussing CF also discussed other morphologic features of the embryo, studies that mention embryo morphology, grade or quality were also included. Articles that looked at non-human embryo fragmentation, case reports, case series, book chapters and review papers were excluded. Titles and abstracts were screened, and study quality and bias were assessed. The primary outcomes of interest were embryo quality, blastocyst formation, and pregnancy outcome.

## Results

**Fig. 1:**
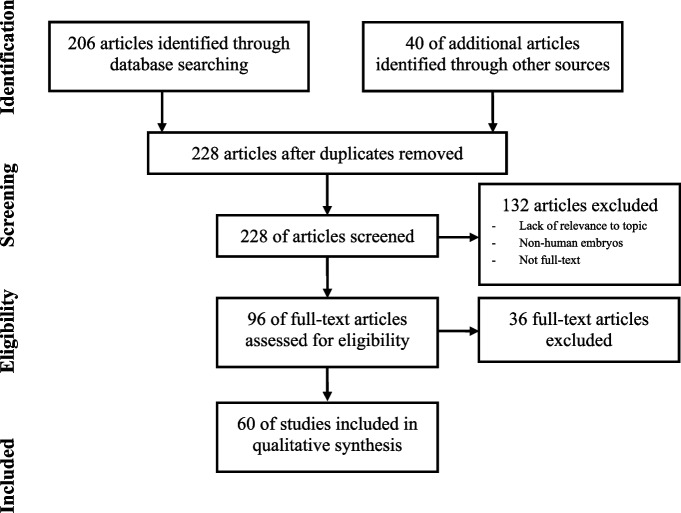
Article Identification and Screening

Figure 1 provides details of study screening and inclusion. There were 206 studies screened between the two search engines PubMed (*n*=106) and Google Scholar (*n*=100). There were 18 duplicates giving a total of 188 articles. Due to the small number of studies from the search criteria, no filter of time was placed. After removal of non-full text articles, articles that used non-human embryos, and articles not relevant to the topic, 20 articles were eligible for inclusion. Forty relevant references from the articles were also extracted, reviewed, and included in this review. These additional articles were reviewed with the same inclusion and exclusion criteria as mentioned above. A total of 60 articles were included in the qualitative synthesis of this review.

### Origin and etiology of CF

**Fig. 2:**
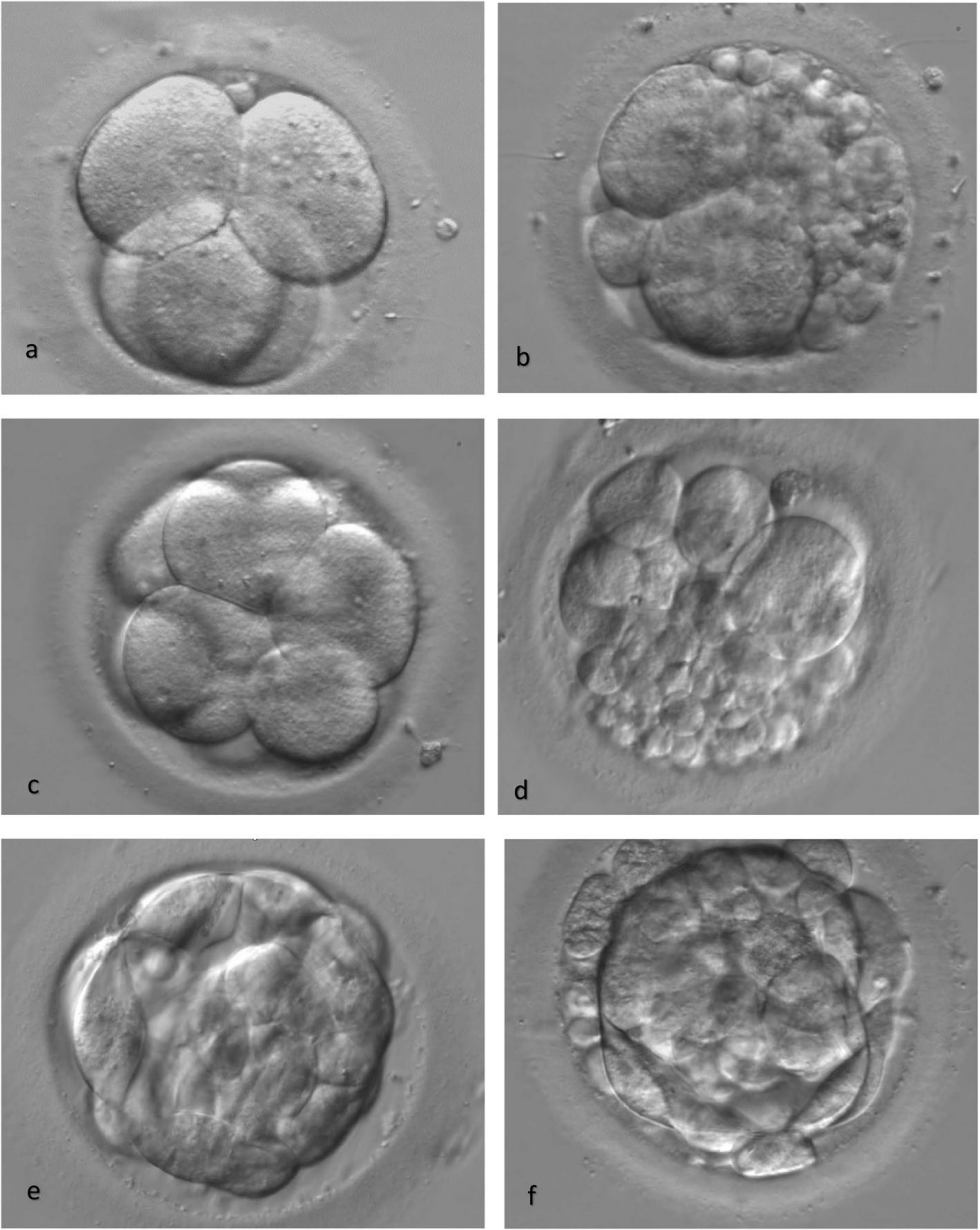
Human cleavage stage embryos a) Day-2 embryo at 4-cell stage with no fragmentation, b) fragmented Day-2 embryo, c) Day-3 embryo at 8-cell stage with no fragmentation, d) fragmented Day-3 embryo, e) Day-5 cavitating Morula with no fragmentation, f) fragmented Day-5 cavitating Morula

**Fig. 3:**
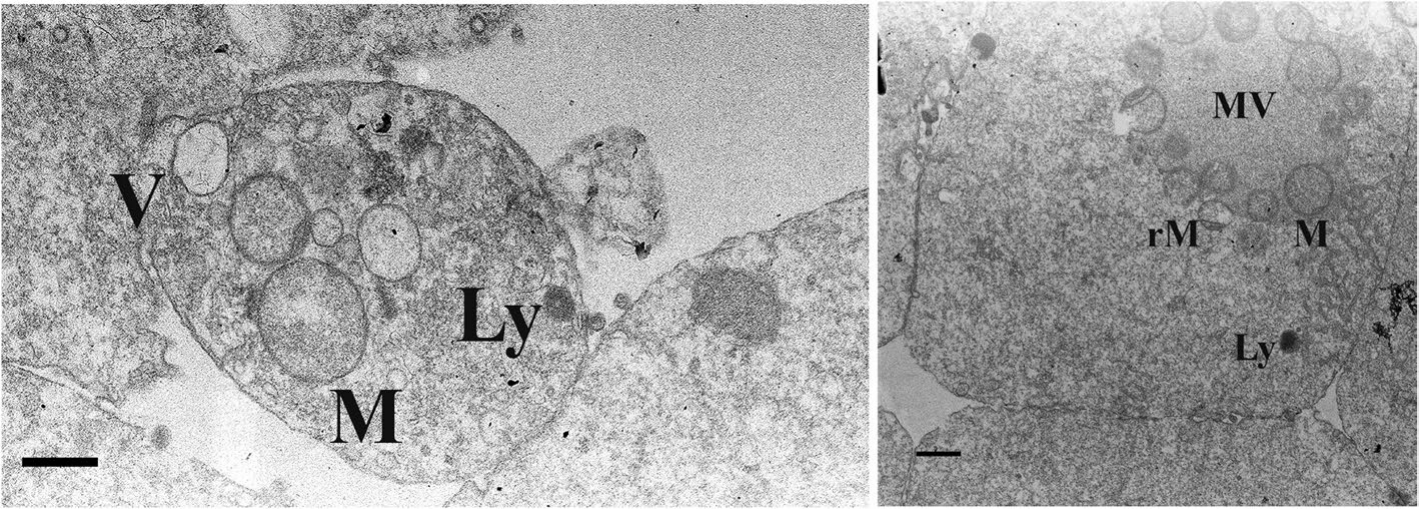
Ultrastructure and organelle microtopography of an embryo fragment by transmission electron microscopy. Ly: primary lysosome, M: mitochondrion, rM: remnant of regressing mitochondrion, MV: mitochondria-vesicle complex, V: vesicle; scale bar: 1 µM

The etiology of CF is not completely understood. There are several proposed theories as to why embryos display variable degrees of fragmentation. Fragmentation has been shown to be a natural, unpredictable process both in vitro and in vivo and is documented in various species [35, 36]. This suggests that embryo fragmentation is neither species-specific nor solely a byproduct of in vitro culture. Assisted reproductive technology (ART) and IVF techniques, such as time-lapse microscopy (TLM) and transmission electron microscopic (TEM) analyses, have recently allowed for further understanding of embryo developmental potential and fragmentation (Figs. 2 and 3). Seven of the included studies in this review propose potential hypotheses as to the origin of CF (Table 1). Three of the articles evaluated gamete quality as related to fragmentation in a developing embryo [37–39].

**Table 1:** Studies examining the origin of cytoplasmic fragmentation in human embryos

**Author**	**Year**	**Title of Paper**	**Type of study**	**No. of study participants**	**Embryo stage**	**Age**	**Intervention (stim protocol)**	**Outcome measure**
Meseguer et al.	2008	The significance of sperm DNA oxidation in embryo development and reproductive outcome in an oocyte donation program: a new model to study a male infertility prognostic factor	Prospective	Infertile male partners from couples undergoing oocyte donation cycles (*n*=38); 76 semen aliquots analyzed before and after semen processing by swim up; 19 oocyte donors were used for two different recipients (*n*=38 recipients).	Day-2 and 3 embryos	Age range 25 - 49 years, median 39.8 years (SD 5.8 years) in women. Median 40.2 years (SD 6.3 years) in men.	GnRH agonist protocol on oocyte donor (for experiment used pairs of oocyte donation cycles i.e. the same oocyte donor was used with use of two distinct male patient sperm samples).	Sperm DNA oxidation by flow cytometry using the OxiDNA assay and correlated it with embryo quality parameters, implantation, and pregnancy outcome.
Stensen et al.	2015	Fragmentation of human cleavage-stage embryos is related to the progression through meiotic and mitotic cell cycles	Observational	1,943 oocytes from 297 patients and 372 embryos from 100 patients.	ICSI performed 36-41 hours after hCG. Percent of fragmentation 42-45 hrs after insemination. Embryo transfer performed on day 2 after oocyte retrieval.		Controlled ovarian stimulation by luteal-phase GnRH agonist down-regulation protocol and daily injections of either recombinant FSH or purified urinary gonadotropin. Final follicular maturation was induced with 6,500 IU recombinant hCG.	Appearance of meiotic spindle; duration of first, second, and third mitosis.
Ebner et al.	2002	First polar body morphology and blastocyst formation rate in ICSI patients	Prospective	60 consecutive ICSI patients.	Day-5 embryos.	Patient mean age 32.1 +/- 3.8 years.	Long protocol: GnRH agonist and individually adjusted dose of hMG. 5000-10,000 IU of hCG administered to induce ovulation.	First polar body morphology was analyzed to fertilization, embryo quality and blastocyst formation.
Van Blerkom et al.	2000	Differential mitochondrial distribution in human pronuclear embryos leads to disproportionate inheritance between blastomeres: relationship to microtubular organization, ATP content and competence	Observational	29 pronuclear oocytes (17 2PN, 12 3PN).	Pronuclear oocytes and cleavage stage embryos. No embryos involved male factor.	Female age range 25-37 years.	Conventional IVF after GnRH suppression and ovarian suppression and ovulation induction.	Distribution of mitochondria, measurements of the net ATP content of individual blastomeres or anti-tubulin immunofluorescence to determine the relationship between mitochondrial distribution and microtubular organization.
Van Blerkom et al.	2001	A microscopic and biochemical study of fragmentation phenotypes in stage-appropriate human embryos	Observational	91 embryos (50 monospermic, 41 dispermic).	Fragmented monospermic and dispermic pronuclear to early cleavage stage human embryos classified as stage-appropriate during the first 3.5 days of culture.	Female age range 28 - 37 years.	Ovarian stimulation protocol previously described by Van Blerkom et al., 1995.^1^	Temporal, spatial, fine structural, and biochemical aspects of fragmentation were examined.
Hardarson et al.	2002	Internalization of cellular fragments in a human embryo: time-lapse recordings	Observational	Time lapse of one embryo.	First cell cleavage stage embryo.			Internalization of fragmentation.
Sermondade et al.	2012	Characterization of a recurrent poor-quality embryo morphology phenotype and zygote transfer as a rescue strategy	Prospective	53 patients w/ recurrent IVF failures (a mean of 2.8 +/- 1.0 previous unsuccessful IVF cycles characterized by consistent failure of embryonic implantation with high rate of highly fragmented embryos) 53 matched controls had mean of 2.5 +/- 0.7 previous IVF attempts.	Transfer at day-2 or day-3.	Patient mean age 34.7 +/- 4.4 years.	Standard ovarian simulation protocols per Antoine et al., 1997.^2^	Pregnancy rate, delivery rate.

An early study showed that sperm DNA oxidation has been associated with embryo development and quality, and therefore linked to CF [37]. Nucleolar asynchrony in the zygote from sperm DNA fragmentation has previously been shown to predict future low-quality blastocyst development. A positive correlation has also been found between the percentage of sperm OxiDNA-stained cells with embryo fragmentation on day-2 and -3 of development. Sperm DNA oxidation may therefore be associated with fragmented, nonviable, poor-quality embryos [37] . A recent study also showed the negative correlation between sperm DNA fragmentation and blastomere DNA fragmentation and blastulation rate [40]. Further studies are needed to confirm the impact of sperm DNA oxidation on embryo fragmentation.

An observational study documented the degree of fragmentation of human embryos as they progressed through mitotic cell cycles [38]. In this study, the authors analyzed nearly 2,000 oocytes and 372 embryos, and found that increased embryo fragmentation (>50%) was associated with a specific pattern of development: delayed first division (oocyte spindle detected at 36.2 hours after hCG injection vs. 35.5 hours in low fragmentation), a significantly earlier start of the second mitosis (8.9 hours vs. 10.8 hours after the first mitosis), and a significant delay of the third mitosis after the second mitosis (2.2. hours vs. 0.6 hours). The authors did not comment on whether fragmentation could be a result of the cell dividing before proper chromosome alignment, or if existing aneuploidy resulted in erroneous cleavage patterns [38].

Polar body (PB) fragmentation has also been investigated in relation to cytoplasmic fragmentation. Ebner et al., in a prospective study analyzed the relationship between a fragmented first PB and embryo quality in patients undergoing ICSI. Two groups of oocytes were analyzed according to PB fragmentation: intact first PBs and those with fragmented PBs. Forty-two hours after ICSI, embryo morphology (i.e., number of blastomeres and degree of fragmentation) was recorded. Overall, a significantly higher percentage of cytoplasmic fragmentation was seen in day-2 embryos that originated from oocytes with fragmented first PBs than those with intact PBs (*P* < 0.05). This study further supports the concept that oocyte quality contributes to overall embryo fragmentation and provides evidence that preselection of oocytes may contribute to the prognosis of embryo quality and blastocyst development [39]. The role of PB fragmentation on embryo quality was confirmed in other studies [41, 42], however, a recent study has not recommended considering PB status as a tool for embryo selection [43].

Beyond analysis of gamete quality, other studies have shown a biochemical relationship between embryo competence and fragmentation. One study showed that disturbances in E-cadherin, a cell adhesion protein that plays a critical role in morphogenesis, occur in embryos with cleavage abnormalities and extensive cytoplasmic fragmentation, suggesting a possible mechanism to the loss of embryonic viability [44]. Further, by using mitochondrial fluorescence techniques, Van Blerkom et al., found that mitochondrial distribution at the pronuclear stage may be an epigenetic factor related to the organization of the embryo and further embryonic development [45]. Blastomeres that were deficient in mitochondria and thus ATP at the first or second cell division remained undivided and often died during subsequent culture. Although this study examined morphologically normal (unfragmented) cleavage-stage embryos, it may support the idea that perinuclear mitochondrial distribution and microtubular organization influence developmental capacity of early cleavage-stage embryos [45]. Higher numbers of mitochondria reported in fragmented compared to the normal blastomeres show the rapid depletion of ATP in the fragmented embryos [21]. There have also been reports of increased gene transcription of mitochondrial factors like OXPHOS complexes, ATP synthase, and mtDNA content in highly fragmented embryos compared to controls [46]. Mitochondrial activity is lower and more centralized in fragmented embryos compared to good quality embryos on day-3 [47]. Mitochondria are the main source of ATP for embryo mitosis, and their proper function is essential for embryo development. More research is needed to elucidate the morphology and role of mitochondria in embryo development, especially in relation to fragmentation.

A subsequent study by Van Blerkom et al., analyzed the temporal and spatial aspects of fragmentation through TLM and TEM analyses from the pronuclear to the 10-12-cell stage. Through TLM, the authors visualized the non-discrete, dynamic nature of fragments and noted that many were “bleb-elaborations” of the plasma membrane and cytoplasm. They characterized two patterns of fragmentation: definitive and pseudo-fragmentation. Definitive fragmentation was described as fragments detached from a blastomere, and pseudo-fragmentation was assigned when the fragments were no longer detectable during subsequent development. Often one developing embryo would show both fragmentation patterns at different stages of development, suggesting that these patterns may have different etiologies and effects on embryo development competence [47]. Hardarson et al., similarly used TLM to document that fragments are dynamic and can be internalized throughout cleavage during culture periods. The contents of the fragments were noted to be internalized and released into the cytoplasm of the blastomere and seen on multiple time-lapse photographs as a cytoplasmic turbulence. This is the first reported evidence that cellular fragments can “disappear” during the culture period in human IVF [26]. It seems that in mild to moderate CF, the timing of embryo evaluation and grading can affect the reported percent of fragmentation.

Lastly, we have included a preliminary study performed by Sermondade et al., that suggests a specific subgroup of patients who have had repeated IVF failures (presumably due to a recurring high rate of fragmented embryos) may benefit from early intrauterine embryo transfer at the zygote stage (2PN) [48]. Data showed a delivery rate per oocyte retrieval of 18.9%, which was significantly higher than the delivery rate of 7.5% in the matched control group. The results were encouraging and suggestive of a safe, non-invasive rescue strategy for patients who experience recurrent highly fragmented embryos and failed IVF attempts. The data further suggests that fertilized oocytes of this subgroup may have deficiencies in certain maternal factors (i.e., stress-response factors) that do not allow normal embryo development in culture environments [48]. Another study was also confirmed application of zygote transfer in patients with history of low-quality embryos [49]. However, further studies are required to verify the impact of this technique for patients with history of fragmented embryos.

Apoptosis is another proposed etiology of fragmentation. Apoptosis may occur in blastomeres with defective cytoplasm or abnormal chromosomes, leading to embryo fragmentation [50]. There are several studies reporting apoptosis in both fragments and neighboring blastomeres in a fragmented embryo [24, 50]. Chi et al., showed that fragments are associated with both apoptosis and necrosis [21]. One of the factors that appears to induce apoptosis in blastomeres is suboptimal culture conditions such as hypoxia [51]. In addition, there are controversial reports on the role of reactive oxygen species (ROS) in embryo fragmentation [52, 53]. It has been shown that ROS are present at high levels in the culture media of fragmented embryos [52, 54]. Chen et al., recently showed that embryo culture in 5% oxygen, from days 1 to 3, is associated with higher embryo quality and live birth rate compared to 20% oxygen [55]. The effects of culture condition modifications, such as hypoxia and ROS, on embryo fragmentation need to be clarified to understand the importance of culture condition in this process.

Membrane compartmentalization of DNA, abnormal cytokinesis, and extra vesicular formation are other proposed theories for embryo fragmentation [8]. Defects or damages in mitochondria are associated with low ATP and high ROS production leading to a compromised cell division and cytokinesis [27]. In addition, there is a correlation between embryo fragmentation and ploidy status. Chavez et al., showed that CF was seen in a high proportion of aneuploid embryos, and that meiotic and mitotic errors may cause fragmentation in different cell development stages. Meiotic errors were associated with fragmentation at one-cell stage while mitotic errors were associated with fragmentation at interphase or after first cytokinesis [56]. Chromosomally abnormal embryos often have severe fragmentation, which may be another cause of CF [55, 57].

Overall, the precise cause of CF has yet to be clearly defined. The above investigations have elucidated potential sources and associations of what is likely a complex and multifactorial process and represent our current understanding of CF origin.

### What is contained in CF?

Four of the included studies used various technological advances to study the contents of CF in human embryos (Table 2). Two studies used TEM methods to evaluate fragment ultrastructure (Fig. 3) [21, 58]. Fragments were extracted from embryos with 10-50% fragmentation and the ultrastructure evaluated by TEM. Micrographs showed that the fragments had a distinct membrane containing cytoplasmic organelles including mitochondria, mitochondria-vesicle complexes, Golgi apparatus, primary lysosomes, and vacuoles. Mitochondria were the most abundant structure.

**Table 2:** Studies examining the content of cytoplasmic fragments

**Author**	**Year**	**Title of Paper**	**Type of study**	**No. of study participants**	**Embryo stage**	**Age**	**Intervention (stim protocol)**	**Outcome measure**
Halvaei et al.	2016	Ultrastructure of cytoplasmic fragments in human cleavage stage embryos	Prospective	150 ICSI cycles with male factor infertility.	Cleavage-stage embryos grades B and C (10-50% fragmentation).	Females <39 years.		Evaluate ultrastructure of cytoplasmic fragment and perivitelline coarse granulation removal (cosmetic microsurgery) from embryos before embryo transfer on ART outcomes (rates of clinical pregnancy, live birth, miscarriage, multiple pregnancies, and congenital anomaly).
Halvaei et al.	2016	A novel method for transmission electron microscopy study of cytoplasmic fragments from preimplantation human embryos	Observational					Explore intracellular damage and organelle distribution - aims to introduce new method for ultrastructural evaluation of fragments without damaging human cleaving embryos.
Johansson et al.	2003	There is a cutoff limit in diameter between a blastomere and a small anucleate fragment	Retrospective	44 pre-embryos; 358 analyzed cells.	Pre-embryos which underwent ICSI or regular IVF program then graded on embryo Day-2 or 3.		Short-acting gonadotropin-releasing hormone agonist preparation followed by stimulation with recombinant FSH.	DNA content of cells of various sizes from early human pre-embryos.
Chi et al.	2011	Fragmentation of embryos is associated with both necrosis and apoptosis	Prospective	IVF patients (*n*=3) donated *n*=16 frozen embryos.	Fragment removal on Day-2 embryos.	Patient ages 24, 28, 29 years.		Staining with annexin V (a marker of apoptosis) and propidium iodide (PI, a marker of necrosis), DNA integrity and mitochondrial distribution, and a beneficial effect of fragment removal in human fragmented embryos.

In an additional evaluation of CF contents, Johansson et al., analyzed DNA content of fragments to define a cutoff diameter for an anucleate fragment or blastomere. Findings showed that 98% of fragments <45 µm on day-2 and 97% of those <40 µm on day-3 contained no DNA and, if not reabsorbed into a blastomere, showed a loss of cytoplasm. Presence of essential blastomere organelles such as mitochondria, mRNA, and proteins within cytoplasmic fragments were related to embryo development arrest [59]. Lastly, Chi et al., also used TEM to examine ultrastructure of the human fragmented embryos and found that blastomeres with anucleate fragments contained fewer mitochondria in their cytoplasm compared to normal blastomeres [21].

### Cell death and CF

Eight of the included studies analyzed the relationship between cell death and embryo fragmentation (Table 3). Five studies analyzed the status of chromatin in arrested fragmented embryos through a combined technique for simultaneous nuclear and terminal transferase-mediated DNA end labelling (TUNEL) [24, 60–63]. Two studies used a comet assay to analyze DNA fragmentation [21, 63]. Four of the eight studies used Annexin V staining [21, 61–63] with three including the presence of propidium iodide (PI) to compare apoptosis to necrosis [21, 61, 63].

**Table 3:** Studies examining the relationship between cell death and cytoplasmic fragmentation

**Author**	**Year**	**Title of Paper**	**Type of study**	**No. of study participants**	**Embryo stage**	**Age**	**Intervention (stim protocol)**	**Outcome measure**
Jurisicova and Varmuza	1996	Programmed cell death and human embryo fragmentation	Experimental - Prospective	229 embryos; 203/229 embryos showed various degrees of fragmentation.	Donated spare human preimplantation embryos arrested at different stages of development ranging from 2-cell to uncompacted morulae.		GnRH agonist (Lupron) in long protocol and human menopausal gonadotrophin (HMG).	Determine whether morphological features of apoptosis are observed in fragmented human preimplantation embryos.
Levy et al.	1998	Annexin V labelling and terminal transferase-mediated DNA end labelling (TUNEL) assay in human arrested embryos	Prospective	50 embryos.	Preimplantation embryos (totally fragmented or developmentally arrested); transfers at Day-2 (2-4 cell stage embryos).	Female age range 28 - 38 years.	GnRH agonist in long protocol and human gonadotrophins (FSH).	Annexin V and TUNEL staining, abnormal chromatin and cellular actin cortex (detection of phosphatidylserine exposure was performed using annexin V; the chromosomal breakdown preceding the nuclear collapse of apoptotic nuclei was tested using the terminal transferase-mediated DNA end labelling (TUNEL) assay).
Liu et al.	2000	Expression of apoptosis-related genes in human oocytes and embryos	Prospective		TUNEL labeling: 18 fragmented embryos at the one-cell to six-cell stages (10-70% fragmented), 3 blastocysts (two from 2 pronuclei and one from 3 pronuclei stage), 3 viable embryos, 3 nonfertilized oocytes. ANNEXIN staining: 19 embryos at the one-cell to nine-cell stages (15-70% fragmented), 2 nonfertilized oocytes. RT-PCR: 8 viable embryos at the two-cell to nine-cell stage (0% fragmentation), 17 arrested embryos at the one-cell to four-cell stage (10-50% fragmented), 21 nonviable embryos at the two-cell to five-cell stage (10-90% fragmented) 24 immature oocytes (at the GV stage), 15 nonfertilized oocytes.			DNA fragmentation and phosphatidylserine translocation, two markers for apoptosis; frequency of gene expression in viable embryos, arrested embryos, immature oocytes and non-fertilized oocytes.
Chi et al.	2011	Fragmentation of embryos is associated with both necrosis and apoptosis	Prospective	IVF patients (*n*=3) donated *n*=16 frozen embryos.	Fragment removal on Day-2 embryos.	Patient ages 24, 28, 29 years.		Staining with annexin V (a marker of apoptosis) and propidium iodide (PI, a marker of necrosis), DNA integrity and mitochondrial distribution, and a beneficial effect of fragment removal in human fragmented embryos.
Metcalfe et al.	2004	Expression of 11 members of the BCL-2 family of apoptosis regulatory molecules during human preimplantation embryo development and fragmentation	Retrospective		Representative embryo panel: pronucleate to 8-cell stage embryo. Fragmented embryo panel: early cleavage embryos.			Expression of 11 BCL-2 genes in individual human embryos of normal development and in severely fragmented embryos.
Van Blerkom et al.	2001	A microscopic and biochemical study of fragmentation phenotypes in stage-appropriate human embryos.	Prospective	72 day 3 embryos transferred.	Annexin V staining: *n*=23 embryos; TUNEL analysis: 41 8-12 cell, day 3.0-3.5 embryos where fragment clusters that arose during early cleavage were either no longer apparent (*n*=14) or were still detectable to different extents (*n*=27). 'Comet' assay: 8-12 cell stage embryos (monospermic, *n*=6; dispermic, *n*=17) in which fragments occurred during the 2-4 cell stage were still detectable.	Female age range 28-37 years.	Ovarian stimulation protocol previously described by Van Blerkom et al., 1995.^1^	Temporal, spatial, fine structural, and biochemical aspects of fragmentation were examined.
Bencomo et al.	2006	Apoptosis of cultured granulosa-lutein cells is reduced by insulin-like growth factor I and may correlate with embryo fragmentation and pregnancy rate.	Prospective	81 patients; 92 IVF cycles. Indications for IVF: tubal infertility (9 patients, 20 cycles), male infertility (36 patients, 40 cycles), endometriosis (7 patients, 9 cycles), PCOS (13 patients, 13 cycles), unknown factor (3 patients, 4 cycles).	Day-2 embryos; looked at apoptosis in GL cells.	Mean age 34.7 +/- 5.2 years (range, 23 - 45 years).	Ovulation induction by recombinant FSH combined with recombinant LH or hMG. Ultrasound- guided follicle retrieval performed 36 hours after administration of hCG. ICSI was performed in all cycles.	Detection of apoptosis by using caspACE FITC-VAD-FMK, a fluorescent in situ marker for activated caspases; embryo fragmentation; and pregnancy.
Antczak and Van Blerkom	1999	Temporal and spatial aspects of developmental potential of the embryo itself. In-vitro study of fragmentation in early human embryos: possible effects on developmental competence and association with the differential elimination of regulatory FP and developmental events, such as ability to blastulate, proteins from polarized domains	Prospective	2293 fertilized eggs from 257 IVF cycles.	First 72 hrs of human embryo culture.	Mean age 36 years (range, 23 - 41 years).	Ovarian stimulation protocol previously described by Van Blerkom et al., 1995.^1^	Effect of fragmentation on the distribution of the following eight regulatory proteins found to be: (i) localized in the mature oocyte in subplasmalemmal, polarized domains; and (ii) unequally inherited by the blastomeres during cleavage: leptin, signal transducer and activator of transcription 3 (STAT3), Bax, Bcl-x, transforming growth factor beta 2 (TGF beta 2), vascular endothelial growth factor (VEGF), c-kit and epidermal growth factor R (EGF-R).

Jurisicova et al., used a combined nuclear and fragmented DNA labeling approach which allowed distinction between chromatin status and DNA fragmentation, which serve as markers of apoptosis versus necrosis respectively [60]. After fertilization, embryos were stained with 4,6-diamidino-2-phenylindole (DAPI). In cases of compromised cell membrane integrity, DAPI stain was observed in the cytoplasm as a sign of necrosis. Concomitant use of TUNEL labeling reflected the integrity of the DNA and allowed distinction between necrotic and apoptotic cells. Through combined techniques of DAPI/TUNEL, TEM, scanning electron microscopy (SEM) and stereomicroscopic observations, 153 of 203 (75.4%) fragmented early cleavage-stage embryos displayed signs of apoptosis (i.e., chromatin condensation, cellular shrinkage, DNA fragmentation, presence of cell corpses) with or without normal nuclei [60].

Similarly, Levy et al., analyzed early arrested or fragmented preimplantation embryos and the pattern of DNA fragmentation using TUNEL assay and the presence of phosphatidylserine through Fluorescein isothiocyanate (FITC)-labelled Annexin V, a phosphatidylserine binding protein. The authors observed TUNEL staining in one or more nuclei of 15 out of 50 (30%) arrested embryos from the 2-cell stage to uncompacted morulae, all of which had high degrees of CF. Furthermore, embryos with regular-sized blastomeres without fragmentation were all TUNEL negative [50].

A separate prospective study by Antczak et al., explored the possible association between fragmentation and apoptosis using PI and Annexin V staining of plasma membrane phosphatidylserine and TUNEL analysis of blastomere DNA [24]. In contradistinction to prior studies, these authors found no direct correlation between fragmentation and apoptosis. Virtually all blastomeres that were PI negative, intact or fragmented, showed no TUNEL or annexin V fluorescence, suggesting no signs of apoptosis [24].

Liu et al., used a similar methodology of TUNEL labeling and Annexin V staining to detect markers of apoptosis in fragmented human embryos derived from IVF [61]. Overall, highly fragmented embryos had apoptotic features including bright fluorescence (positive TUNEL labeling signifying DNA fragmentation) on the cell corpses and in intact blastomeres [61]. By staining cells with both annexin V and PI, this study was able to demonstrate that apoptosis occurs frequently in fragmented human embryos and the coexistence of apoptotic, necrotic and viable sibling blastomeres can occur. Sibling blastomeres within an embryo often showed apoptotic features that led to secondary necrosis while others did not initiate apoptosis. The authors did not find a significant difference in the expression frequency of apoptotic genes between viable and nonviable or arrested embryos [61].

Chi et al., stained human embryos (*n*=10) with annexin V and PI and found that human fragmented embryos exhibited characteristics of both necrosis and apoptosis [20]. Rather than TUNEL assay, these authors used a modified sperm comet assay to investigate DNA fragmentation of human fragmented embryos. They found that 6/7 human fragmented embryos (85.1%) stained positively for PI with the intensity of staining increasing with the degree of fragmentation. Of note, DNA fragmentation was observed in fragmented human embryos but not in the normal embryo [21].

Metcalfe et al., analyzed the expression of 11 BCL-2 family genes in normally developing embryos and in severely fragmented embryos [64]. They found that the expression of BCL-2 family genes was highest in the pronuclear stage and eight-cell stages, and lowest at the two-cell, four-cell, and blastocyst stages in developmentally intact embryos. Furthermore, the expression did not change in fragmented embryos, suggesting that embryo fragmentation does not likely compromise mRNA integrity and gene detection [64]. However, like Liu et al., [61] these authors did detect far fewer pro-apoptotic BCL-2 genes in fragmented embryos at the eight-cell stage. The authors noted that these findings do not distinguish between iatrogenic apoptosis from suboptimal in-vitro culture conditions [64]. A separate study by Jurisicova et al. similarly analyzed gene expression at the 2-, 4- and 8-cell stage of fragmented embryos. Embryos that had 30-50% fragmentation showed a significant increase in Hrk mRNA levels, a BCL-2 protein encoding gene (*P* = 0.016). Further, these authors found an increase in Caspase-3 mRNA in fragmented embryos, as well as induction of Caspase-3-like enzyme activity in nucleated fragments, although this finding was not statistically significant [65].

Van Blerkom et al., also used TUNEL assay in conjunction with the comet assay as a method of identifying the specific pattern of cell death (necrosis, lysis or apoptosis) and the extent of DNA damage in developing embryos [47]. They analyzed the integrity of the plasma membrane through annexin V staining with PI. They examined both transient and persistent fragment clusters at day-3 and 3.5 embryos for evidence of programed cell death using time-lapse video and TEM. In contrast to previous studies, they found no indication of nuclear DNA damage or loss of membrane integrity. These results, led the authors to hypothesize that the fragmentation observed was not characteristic of programed cell death, but rather resembled features of oncosis. The culture in this study was not severely oxygen-deprived and thus the authors concluded that this oncosis-like process was potentially a result of disproportionate mitochondrial segregation during the first cleavage division. Without sufficient mitochondria, the early blastomeres did not maintain adequate ATP for normal cell function which may have precipitated an ATP-driven oncosis-like process [47].

Lastly, a study by Bencomo et al., found correlations between the degree of apoptosis in human granulosa-lutein (GL) cells, the outcome of IVF-ET cycle, the percentage of embryo fragmentation, and patient’s age [66]. Human GL cells were collected from follicular fluid, cultured for 48 hours, and marked with caspACE FITC-VAD-FMK, a fluorescent marker for activated caspases. Results showed that GL cells of older women (>38 years old) were significantly more susceptible to apoptosis at 43.2 ± 18.0% compared to the younger group (<38 years old) with a mean percentage of apoptotic cells 33 ± 17.2%. Women who had a positive pregnancy had a lower level of apoptosis in GL cultures than those who did not get pregnant (30.2 ± 14% vs. 40.4 ± 19.5%). There was a positive correlation between embryo fragmentation and GL cell apoptosis (*r* = 0.214). Overall, the level of apoptosis of cultured GL cells was correlated with IVF outcome [66].

These studies demonstrate the diversity among techniques to evaluate cell death in the developing embryo. TUNEL labeling, sperm comet assay, annexin V staining or some combination of these techniques have been described. Furthermore, there are discrepancies between the stage at which apoptosis might occur, with majority of studies cited here suggesting that cell death occurs in early stages of development before blastocyst formation. While some studies suggest that fragmented embryos display signs of apoptosis, these findings are still disputed and the distinction between apoptosis and necrosis is not clearly defined in the literature.

### Patient age and CF

There are inconsistencies within the literature regarding the relationship between maternal age and CF. A total of six studies in this review focused on this relationship (Table 4). Three of the studies found a positive correlation between patient age and degree of embryo fragmentation [67–69]. The other three studies found no age-related correlation between embryo fragmentation or quality [7, 70, 71].

**Table 4:** The relationship between patient age and cytoplasmic fragmentation

**Author**	**Year**	**Title of Paper**	**Type of study**	**No. of study participants**	**Embryo stage**	**Age**	**Intervention (stim protocol)**	**Outcome measure**
Ziebe et al.	2001	Embryo quality and developmental potential is compromised by age	Retrospective	878 IVF cycle transfers of 1683 fresh embryos, single transfers (*n*=292), dual transfers (*n*=366), triple transfers (*n*=220); all transferred embryos in each cycle were of identical quality score and cleavage stage.	Cleavage stage embryos.	Mean age 33.1 years (range, 20-45 years).	Ovarian stimulation induced by hMG. HcG was injected 36 h before oocyte retrieval.	Quality and developmental potential of oocytes and embryos in ART program.
Keltz et al.	2006	Predictors of embryo fragmentation and outcome after fragment removal in in vitro fertilization	Retrospective	327 nondonor, fresh IVF cycles.	Assisted hatching and fragment removal on Day-3 embryos.	Patient age range 34.7 +/- 4.5 to 37 +/- 4.6 years.	Combination GnRH agonists and gonadotropins in a long or short protocol combined with 10,000 IU hCG. Oocyte retrieval 35 hours afer hCG.	Predictors of fragmentation. Evaluated age, basal FSH and E(2) levels, the number of retrieved oocytes, and fertilization rates. Outcome assessments following defragmentation included rates of implantation, clinical pregnancy, spontaneous abortion, and live birth.
Alikani et al.	1999	Human embryo fragmentation in vitro and its implications for pregnancy and implantation	Retrospective	2,410 total procedures; 1,727 procedures were found to be homogenous for degree of fragmentation ("W" groups); 570 procedures were found to be homogenous for pattern of fragmentation ("T" groups).	Day-3 embryos.	Mean age 35.7 +/- 4.25 years.	Standard down-regulation protocol or a modification of the microflare protocol (for patients ≥40 and those patients in whom previous response to down-regulation was unsatisfactory).	Degree and pattern of fragmentation on pregnancy and implantation.
Stensen et al.	2010	Routine morphological scoring systems in assisted reproduction treatment fail to reflect age-related impairment of oocyte and embryo quality	Retrospective	4587 couples undergoing IVF (*n*=3073) or ICSI (*n*=1514). > 43,000 oocytes, 25,000 embryos and 7,900 transferred embryos.	Day-2 and day-3 embryos.	Mean age 33 +/- 4.0 years. Five age groups: <25 years, 26-30 years, 31-35 years, 36-40 years and >41 years.	Administration of GnRH agonist in the mid-luteal phase of the preceding cycle during a down-regulation long protocol (*n*=4540) or on day 1 of the follicular phase according to a flare up-protocol (*n*=47). Ovarian stimulation with daily injections of human recombinant FSH or Menopur. Final follicular maturation was induced with 10,000 IU HCG or with 6500 IU HCG. Oocytes retrieved 34-38 hrs later by vaginal route and under US-guidance (*n*=43,632 oocytes).	Number of oocytes retrieved, oocyte quality, maturity, fertilization rates, embryo quality (morphological features), treatment outcome.
Wu et al.	2011	Age does not influence the effect of embryo fragmentation on successful blastocyst development	Retrospective review of patient and cycle data	904 cleavage-stage embryos with normal cellular division; 285 IVF cycles in 276 patient.s	Day-3 embryos. Distribution of embryos: 158 embryos (fragmentation score of 1), 328 embryos (score 2), 202 embryos (score 3), 216 embryos (score 4).	Mean age 30.6 +/- 4.6 years (range, 20 - 44 years).	Ovarian stimulation with either luteal-phase GnRH agonist protocol, GnRH antagonist protocol, or a micro-dose flare protocol. US-guided oocyte retrieval performed 35 hours after administration of either 500 μg of recombinant hCG or 7,500–10,000 IU hCG.	Rate of blastocyst formation as a function of fragmentation on day 3 and maternal age
Lahav-Baratz et al.	2023	Evaluation of fragmented embryos implantation potential using time-lapse technology	Retrospective	379 embryos with more than 5% fragmentation	Day 5 embryos	patients ˃35 compared to ≤35 years old	All women were treated with either a gonadotropin releasing hormone (GnRH) agonist or antagonist protocol.	Implantation potential of fragmented embryos

A retrospective study by Ziebe et al., compared the relationship between age of women undergoing IVF and the proportion of anucleate fragmentation in cleavage-stage embryos. Using a logistic regression analysis, the authors compared the percentage of transfers using fragmented embryos with age; the odds of fragmentation increased by 3% per year (OR 1.033 [95% CI 0.996, 1.071]). There was a linear relationship between age and embryo fragmentation rate, with an increase in fragmentation of 0.76% per year (95% CI -0.09%, 1.61%) [68].

Keltz et al., assessed various predictors of embryo fragmentation in IVF and found that increased maternal age and lower number of oocytes and embryos were associated with increased embryo fragmentation. There was a significant difference between cycles with fragmented embryos (*n*=74) at a mean age of 36.9 ± 4.24 years as compared to cycles with no fragmented embryos (*n*=234) at a mean age of 35.4 ± 4.74 years. Overall, this retrospective analysis of fresh IVF cycles found that embryo fragmentation is indeed associated with older age and ultimately poor cycle outcome [67].

Contrary to these findings, an early study by Alikani et al., showed no relationship between maternal age and CF [7]. In a retrospective analysis of degree and pattern of embryo fragmentation on days 2 and 3, they defined five patterns of fragmentation. Both the degree and pattern of fragmentation impacted pregnancy and implantation rate, but the authors found no correlation between appearance of any CF pattern and maternal age. The average maternal age in their population was 35.7 ± 4.25 years [7]. Another study by Stensen et al., analyzed the effect of chronological age on oocyte quality (assessed by maturity) and embryo quality (assessed by cleavage-stage, blastomere size and embryo fragmentation). Women were divided into five age groups: ≤25, 26–30, 31–35, 36–40 and ≥41 years. The embryo morphological score was based on fragmentation and blastomere size with score of 0-4 where score of 4 being equally sized blastomeres and no fragmentation and score of 0 being cleavage arrest or morphologically abnormal embryo. The mean oocyte score and embryo morphology score were not found to be significantly different across the age groups [70]. Wu et al., also showed that age does not influence embryo fragmentation. Patient ages ranged from 20 to 44 years with a mean age of 30.6 ± 4.6 years and were divided into age groups of ≤29, 30–34, 35–37, 38–40, and ≥41 years of age. Analysis of embryos with similar degrees of fragmentation was used to assess whether maternal age was associated with embryo fragmentation and blastocyst development. There was no correlation between age and embryo fragmentation as a continuous variable (*r* = 0.02; *P* = 0.25) nor was there a correlation when age was divided into the groups (*P* = 0.2). They also found that neither age (*r* = -0.08; *P*=0.16) nor degree of fragmentation (*r* = -0.01; *P* = 0.81) had a significant impact on blastocyst development [71].

Recently, a retrospective time-lapse study evaluated the implantation rate of 379 fragmented embryos. The results showed that there was an association between advanced maternal age and fragmentation. Fragmentation rate was higher in patients ˃35 compared to patients ≤35 years old. It seems that the lower quality of oocytes in older patients results in increasing fragmentation [69]. Overall, the included studies have differing conclusions on the effect of maternal age and CF; varying definitions and analysis of CF remain a limitation.

### IVF vs ICSI procedures and CF

Five of the included studies compared embryo quality between conventional IVF and intracytoplasmic sperm injection (ICSI) procedures (Table 5). Two of these studies found that ICSI was associated with impaired embryo morphology compared to IVF [72, 73], while the other three showed no difference in embryo quality between the two fertilization modalities [74–76]. There were no studies within our search that identified embryos created by ICSI having greater morphology grade, or less embryo fragmentation, than IVF.

**Table 5:** Studies which examine the difference between IVF and ICSI procedures and relationship of cytoplasmic fragmentation

**Author**	**Year**	**Title of Paper**	**Type of study**	**No. of study participants**	**Embryo stage**	**Age**	**Intervention (stim protocol)**	**Outcome measure**
Frattarelli et al.	2000	Intracytoplasmic sperm injection increases embryo fragmentation without affecting clinical outcome	Clinical trial	253 patients (71 ICSI, 182 IVF), total of 992 graded embryos compared (289 embryos ICSI, 70 embryos IVF).	Day-3 embryos.	37 years of age or less; mean age for ICSI group 32.7 years (SD = 3.3); mean age for IVF 32.3 years (SD = 3.3).	Stimulation with variety of stim protocols (GnRH analogue) or oral contraceptive pill down regulation followed by stimulation with exogenous gonadotropins or concomitant initiation of GnRH-a and exogenous gonadotropins ("flare" cycles). Transvaginal ultrasound follicular aspiration 34-36 hours after injection with 10,000 units of hCG.	Embryo morphology and degree of fragmentation.
Hsu et al.	1999	Embryo implantation in in vitro fertilization and intracytoplasmic sperm injection: impact of cleavage status, morphology grade, and number of embryos transferred	Retrospective	Total of 422 patients. 211 consecutive couples undergoing ICSI who were matched with 211 couples undergoing IVF therapy during the same time frame. 1,737 embryos transferred available for analysis.	Day-3 embryos.		Controlled ovarian hyperstimulation by combination of GnRH agonist (Lupron) and FSH (Metrodin or Fertinex). Transvaginal follicular aspiration 34-35 hours after hCG administration.	Day-3 embryo quality as judged by the number of blastomeres and morphology scoring.
Garello et al.	1999	Pronuclear orientation, polar body placement, and embryo quality after intracytoplasmic sperm injection and in-vitro fertilization: further evidence for polarity in human oocytes?	Prospective	251 two pronuclear (2PN) embryos (124 ICSI, 127 IVF) from 64 patients studied.	Morphologic grade of day-2 embryos recorded.	IVF: 33.6 ± 5.4 years (range, 24–46 years). ICSI: 33.3 ± 4.3 years (range, 26–45 years). Total: 33.5 ± 5.0 (24–46 years).	GnRH from day 21 of menstrual cycle, 2 day withdrawal bleed by stimulation with FSH, ultrasound guided retrieval 36 hrs after hCG 10,000 U.	Embryo morphology.
Yoeli et al.	2008	Comparison of embryo quality between intracytoplasmic sperm injection and in vitro fertilization in sibling oocytes	Prospective	177 patients, 36 with male-factor infertility and 141 with low fertilization rate in previous standard IVF cycle. Total of 3,006 oocytes were retrieved; mean number of oocytes retrieved per patient was 17.1 +/- 7.2.	Day-2 and day-3 embryos.	Mean age of 31.9 +/- 5.2 years (range, 21 - 44 years).	Stim protocol not described. 10,000 IU dose of hCG injected when at least two follicles reached diameter of 18 mm, US guided follicular aspiration 34-36 hours afer hCG.	Embryo quality.
Ruiz et al.	1997	The role of in vitro fertilization and intracytoplasmic sperm injection in couples with unexplained infertility after failed intrauterine insemination	Prospective	70 couples with unexplained infertility (*n*=63) and mild endometriosis (*n*=7) undergoing IVF after four failed controlled ovarian stimulation and IUI cycles.	Cleavage stage embryos.	Mean age 31.9 +/- 0.4 years.	Protocols for standard IVF and ICSI procedures as documented in previous work.	Fertilization, cleavage, embryo quality in IVF and ICSI inseminated oocytes.

Frattarelli et al., directly examined the effect of ICSI on embryo fragmentation and implantation rate compared to IVF. There was a significant difference in mean embryo grade between IVF and ICSI. IVF patients had significantly more grade I, or non-fragmented, embryos compared to the ICSI group (*P* < 0.01). However, there was no significant difference in mean number of embryos per embryo grade II – IV [72].

Similarly, Hsu et al., compared embryo quality, morphology, and cleavage after ICSI with standard IVF patients. They defined the grading system from 1 – 5, ranging from no fragments (grade 1) to severe or complete fragmentation (grade 5). They found that for the overall population, when comparing ICSI and IVF patients after matching for age and number of embryos transferred, the number of embryos with good morphology was significantly greater in the IVF group compared to ICSI (*P* < 0.006). The average morphology scores, similar to the results of Frattarelli et al., were significantly different between the ICSI group and the IVF group. They also found IVF patients’ embryos to have significantly better cleavage rate than those from ICSI patients (*P* < 0.001) [73].

Garello et al., evaluated if fertilization via ICSI influences pronuclear orientation, PB placement, and embryo quality when compared to IVF. Embryos were assessed using morphology, and grouped as good (grades 1-2), average (grades 3-4), or poor (grades 5-6). Embryos were also assessed for cleavage regularity and proportion of fragmentation (0, <20%, 20–50%, >50%). There was no statistically significant difference in mean morphology (good, average, poor) between the groups, although they did note an apparent increase in grade 4 versus grade 3 embryos after ICSI procedure. The two groups had similar proportions of fragmentation [74].

Two other studies took a unique approach in comparing embryo quality in ICSI and IVF patients by using randomized sibling oocytes [75, 76]. Yoeli et al., studied oocytes retrieved from patients with a less than 40% fertilization rate in a previous standard IVF cycle and divided these oocytes into a conventional insemination group and an ICSI group. Each group had over 1400 oocytes. Overall, there was no significant difference between the IVF and ICSI groups in terms of cleavage rate or rate of high-quality embryos (both Grade A embryos with ≤10% fragmentation and embryos with ≤20% fragmentation) [75]. Ruiz et al., also analyzed sibling oocytes in patients who had failed intrauterine insemination attempts. The authors similarly found no significant difference in fertilization rates and degree of fragmentation between ICSI and standard IVF groups [76]. Most studies included in the search criteria showed that ART techniques such as ICSI do not significantly impact fragmentation rate in developing embryos, suggesting that ICSI is not a significant contributor to poorer outcomes by way of embryo fragmentation. Of note, the timing of cumulus cell denudation after conventional IVF is a matter of debate; none of the included studies in this review performed short-time insemination. In a meta-analysis reviewing denudation times, the number of good quality embryos produced after retaining cumulus cells was similar to those produced after early removal of these cells, suggesting that brief insemination has no impact on CF [77]. Liu et al. also showed that short insemination time is not associated with different outcomes in terms of embryo development [78].

### Effect of CF on embryo development

It is commonly believed that CF has detrimental effects on embryo development. Thirteen of the included studies found a negative effect of CF on embryo development (Table 6). Various approaches have been used to propose a hypothesis as to how increased fragmentation impedes embryo development.

**Table 6:** Studies examining the effect of cytoplasmic fragmentation on embryo development

**Author**	**Year**	**Title of Paper**	**Type of study**	**No. of study participants**	**Embryo stage**	**Age**	**Intervention (stim protocol)**	**Outcome measure**
Van Blerkom et al.	2001	A microscopic and biochemical study of fragmentation phenotypes in stage-appropriate human embryos	Observational	91 embryos (50 monospermic, 41 dispermic).	Fragmented monospermic and dispermic pronuclear to early cleavage stages human embryos classified as stage-appropriate during the first 3.5 days of culture.	Female age range 28 - 37 years.	Ovarian stimulation protocol previously described by Van Blerkom et al., 1995.^1^	Temporal, spatial, fine structural, and biochemical aspects of fragmentation were examined.
Sathananthan et al.	1990	Ultrastructure of preimplantation human embryos co-cultured with human ampullary cells	Observational	15 embryos.	2-cell stage to blastocyst hatching.			Ultrastructure evaluation.
Antczak and Van Blerkom	1999	Temporal and spatial aspects of developmental potential of the embryo itself. In-vitro study of fragmentation in early human embryos: possible effects on developmental competence and association with the differential elimination of regulatory FP and developmental events, such as ability to blastulate, proteins from polarized domains	Prospective	2293 fertilized eggs from 257 IVF cycles.	First 72 hrs of human embryo culture.	Female mean age 36 years (range, 23 - 41 years).	Stimulation protocol previously described by Van Blerkom et al., 1995.^1^	Effect of fragmentation on the distribution of the following eight regulatory proteins found to be: (i) localized in the mature oocyte in subplasmalemmal, polarized domains; and (ii) unequally inherited by the blastomeres during cleavage: leptin, signal transducer and activator of transcription 3 (STAT3), Bax, Bcl-x, transforming growth factor beta 2 (TGF beta 2), vascular endothelial growth factor (VEGF), c-kit and epidermal growth factor R (EGF-R).
Pellestor et al.	1994	Direct assessment of the rate of chromosomal abnormalities in grade IV human embryos produced by in-vitro fertilization procedure	Prospective	411 grade IV embryos from 327 couples participating in IVF,	Cleaved embryos classified 48 hrs after insemination.	Female age range 24 - 43 years. Male age range 25 - 52 years.	Stimulation protocol previously described by Audibert et al., 1989.^2^	Karyotype of grade IV embryos.
Pellestor et al.	1994	Relationship between morphology and chromosomal constitution in human preimplantation embryo	Prospective	462 preimplantation embryos analyzed (426 poor quality and 36 good quality embryos); chromosomal analysis successful in 153 cases.	Embryos examined 24 hrs and 48 hrs after insemination.			Chromosomal status and morphologic quality of preimplantation eggs.
Munne et al.	1995	Embryo morphology, developmental rates and maternal age are correlated with chromosome abnormalities	Prospective	524 monospermic embryos; 283 analyzed with X, Y, 18, 13/21 probes and remainder with X, Y, and 18 probes.	Cleavage-stage embryos.	Maternal age groups used: 20 - 34, 35 - 39, 40 - 47 years.	Per protocol at Center for Reproductive Medicine and Infertility, The New York Hospital- Cornel University Medical College.	Detection of maternal age effect, complete assessment of mosaicism (by analysis of all cells), relationship with embryonic dysmorphism.
Morgan et al.	1995	Use of videocinematography to assess morphological qualities of conventionally cultured and cocultured embryos	Prospective		24-30 h of in-vitro development.		Stimulation protocols previously described by Wiemer et al., 1993.^3^	Effect of conventional and coculture methodologies on morphological parameters in human embryos and to examine clinical pregnancy rate.
Alikani et al.	2000	Cleavage anomalies in early human embryos and survival after prolonged culture in-vitro	Prospective	102 patients who were elected to have a day-5 embryo transfer.	Day-5 embryos.	Mean ages of regular patients 33.89 +/- 3.7 years. Mean ages of oocyte recipients 40.06 +/- 4.21 years.	Standard down-regulation protocol including 1 mg Lupron. Stimulation with recombinant FSH and HCG administration when lead follicle reached 16-17 mm. Oocyte retrieval 36 h after hCG.	Normal blastocyst development in vitro; impact of cleavage rate, fragmentation, and multinucleation on compaction, cavitation, and inner cell mas and trophectoderm formation was assessed.
Hardy et al.	2003	Maintenance of inner cell mass in human blastocysts from fragmented embryos	Retrospective	363 embryos from donated infertile couples undergoing IVF.	Day-2 to day-6 embryos.		Superovulation induced with human menopausal gonadotropin after pituitary suppression by an LH-releasing hormone agonist. 10,000 u of hCG 36 hrs before egg retrieval.	Rates of development and blastocyst formation. The number of cells in the trophectoderm and inner cell mass and the incidence of apoptosis.
Guerif et al.	2007	Limited value of morphological assessment at days 1 and 2 to predict blastocyst development potential: a prospective study based on 4042 embryos	Prospective	4042 embryos	Assessment of day-1 (zygote stage) to day-5/6 (blastocyst stage).	Mean female age 33.9 +/- 4.0 years (range, 25-43 years).	Ovarian stimulation protocol previously described by Guerif et al., 2004.^4^	Pronuclear morphology on day 1, and early cleavage, cell number and fragmentation rate on day 2 were evaluated for each zygote. Blastocyst transfers were analyzed to their implantation ability and early embryo development parameters.
Ivec et al.	2011	Prediction of human blastocyst development from morulas with delayed and/or incomplete compaction	Prospective	Day-4 embryos (*n*=329); Day 5 embryos (*n*=256).	Day-4 or day-5 embryos.	Females < 37 years.	Stim protocols used combination of GnRH agonist/GnRH antagonist and recombinant FSH/hMG. Egg retrieval carried at 35-36 hrs after hCG injection.	Blastocyst development rate, blastocoel expansion rate, and optimal blastocyst rate. In an optimal blastocyst: surface area, trophectoderm cell number, inner cell mass (ICM) surface area, ICM volume and ICM shape.
Hnida et al.	2004	Computer‐controlled, multilevel, morphometric analysis of blastomere size as biomarker of fragmentation and multinuclearity in human embryos	Prospective	63 ICSI patients. Consecutive cohort of 232 human 2-, 3- and 4-cell embryos from patients referred for ICSI treatment.	Cleavage stage embryos.	Mean female age 32.1 years (range, 24 – 39 years).	Long protocol using GnRH agonist for down-regulation and recombinant FSH for ovarian stimulation. HCG was given 36h before oocyte retrieval.	Mean blastomere size, embryonic fragmentation, and multinuclearity.
Sjoblom et al.	2006	Prediction of embryo developmental potential and pregnancy based on early stage morphological characteristics	Retrospective audit of data	268 patients, 357 treatment cycles IVF (*n*=170) and ICSI (*n*=187).	Days 0-2 embryos.		Hormonal treatment regimen, OCP-Long protocol was used: OCPs for 21 days, administration of GnRH agonists (with nafarelin 200 μg intranasal spray twice a day or leuprolide acetate injections 1 mg SC daily for 14 days. Stimulation with recombinant FSH 150-225 IU injected SC daily. 10,000 hCG given when one or more follicles reached size >18 mm, oocyte pickup 36 h later.	The association of blastocyst development and pregnancy with morphological characteristics.

Van Blerkom et al., showed through time-lapse video and TEM that fragments physically impede cell-cell interactions, interfering with compaction, cavitation, and blastocyst formation [63]. In an ultrastructural observational study by Sathananthan et al., 15 embryos were cultured with human ampullary cell lines and TEM used to evaluate embryo development. They noted degeneration of blastomeres, including incomplete incorporation of chromatin into nuclei and formation of micronuclei, which was possibly a consequence of being adjacent to blastomere fragments [79]. A much larger prospective study by Antczak and Van Blerkom analyzed 2293 fertilized eggs from 257 IVF cycles to examine the effect of fragmentation on the distribution of eight regulatory proteins. Fragmentation reduced the volume of cytoplasm and depleted embryos of essential organelles or regulatory proteins, compromising the embryo developmental potential. They also found that specific fragmentation patterns during various stages of embryo development, i.e., 2- and 4-cell stages, were associated with embryo viability and therefore could have clinical application in the selection of embryos for transfer [24]. As previously mentioned, fragmentation may affect compacted/morula and blastocyst quality [80]. Cell exclusion at this stage is due to failure or abnormal expression of proteins involved in compaction [44, 81]. Blastomeres may also irregularly divide, resulting in fragmentation and exclusion from compaction [82], and excluded cells have a high rate of aneuploidy [83]. Blastocyst quality from fully compacted embryos has been reported to be higher than blastocysts with partial compaction [84].

The hypothesis that fragmentation reflects inherent embryogenetic abnormalities, such as aneuploidy, increased mosaicism, or polyploidy, is supported by multiple studies in this review [55, 57, 85]. Morphologically poor-quality embryos, defined by amount of fragmentation, were often found to have concomitant chromosomal abnormalities [57, 85]. Culture environment has also been implicated in presence and degree of fragmentation. For example, Morgan et al., using video-cinematography found that embryos cultured on a monolayer of feeder cells had fewer fragments than did embryos cultured alone [86]. In addition to aneuploidy and external environment, degree of fragmentation also appears to be related to embryo quality. Both Alikani et al., and Hardy et al., have shown that a small degree of fragmentation (<15%) on day-2 embryos did not affect blastocyst formation but increased (> 15%) fragmentation was associated with significantly reduced blastocyst development [23, 87]. Similarly, a prospective study of over 4000 embryos by Guerif et al., showed that the rate of blastocyst formation increased significantly with decreased fragmentation (<20%) on day-2 embryos [32].

A separate study by Ivec et al., graded day-4 and -5 morulae based on the degree of fragmentation (<5%, 5%–20%, or >20%) and compared their blastocyst development rate. They found a negative correlation between degree of fragmentation and clinically usable blastocysts, optimal blastocysts, and those with a hatching zona pellucida. Through logistic regression analysis, they found that with each increase in percentage of fragmentation in morulae, there was a 4% decrease in the odds of hatching (OR: 0.96, 95% CI: 0.95–0.98; *P* < 0.001) and optimal blastocyst formation (OR: 0.96, 95% CI: 0.94–0.97; *P* < 0.001) [88]. It is important to point out that the degree of embryo fragmentation, no matter at what stage of development, is measured subjectively without standardized methods. One study from Hnida et al., included here recognized this limitation and used a computer-controlled system for multilevel embryo morphology analysis [89]. The degree of fragmentation was evaluated based on digital image sequences and correlated to the blastomere size. Fragments were defined to be anucleate with an average diameter of <40 µm. Not surprisingly, the mean blastomere volume decreased significantly with increasing degree of fragmentation (*P* < 0.001). In addition, average blastomere size was significantly affected by the degree of fragmentation and multinuclearity which may function as a biomarker for embryo quality [89]. Furthermore, Sjöblom et al., analyzed the relationship of morphological characteristics to the developmental potential of embryos [90]. These authors, similar to Hnida et al., found that a large cytoplasmic deficit, i.e., blastomeres not filling the space under the zona, was detrimental to blastocyst development (P < 0.044). However, this is the only study in which the extent of CF observed was not significantly associated with blastocyst development [90]. Another study using time-lapse imaging showed an association between cytoplasmic fragments at the two-cell stage and perivitelline threads. Perivitelline threads can be observed as the cytoplasmic membrane withdraws from the zona pellucida during embryo cleavage. Ultimately, the presence of these threads, despite the level of fragmentation, did not affect embryo development [91]. As demonstrated by the studies described here, the degree of CF has a largely negative effect on embryo development.

### Effect of CF on embryo implantation and pregnancy

In addition to evaluating the effect of CF on preimplantation embryo development, it is important to assess the effect of CF on implantation and pregnancy outcomes. Five of the included studies have shown a negative effect of CF on implantation or pregnancy outcome (Table 7). Assuming that increased fragmentation is detrimental to embryo development, implantation, and pregnancy outcome, it is important to understand the embryo scoring system that determines the best embryo for transfer. Giorgetti et al., used single embryo transfers to devise an embryo scoring pattern to best predict successful implantation. Not surprisingly, higher pregnancy rates were observed with embryos that displayed no fragmentation. The authors found that both pregnancy rate and live birth rate were significantly correlated with a 4-point score based on cleavage rate, fragmentation, irregularities displayed, and presence of a 4-cell embryo on day-2 [12].

**Table 7:** Effect of cytoplasmic fragmentation on embryo implantation and pregnancy

**Author**	**Year**	**Title of Paper**	**Type of study**	**No. of study participants**	**Embryo stage**	**Age**	**Intervention (stim protocol)**	**Outcome measure**
Giorgetti et al.	1995	Embryo score to predict implantation after in-vitro fertilization: based on 957 single embryo transfers	Retrospective	957 single embryo transfers.	Cleavage stage embryos.	Used 38 years as a cutoff age for comparison.	Ovarian stimulation using HMG treatment (150-300 IU/day) or 150-300 IU/day Humegon and/or FSH treatment. Oocytes retrieved after 5000 IU HCG.	Implantation, pregnancy rate, delivery rate.
Racowsky et al.	2009	Is there an advantage in scoring early embryos on more than one day?	Retrospective	1595 embryos, 564 resulted from ICSI and 1031 underwent assisted hatching with or without ICSI. 269 transferred alone, 1326 transferred in double embryo transfers.	Day-3 embryo selection.	Total age range 21.6 - 44.8 years. Mean ICSI: 34.18 +/- 4.37 years. Mean IVF: 35.22 +/- 4.36 years.	Controlled ovarian stimulation using protocols previously described in Skiadas et al., 2006.^1^	Embryonic features: No. cells, pronuclei, fragmentation, symmetry, distribution of nuclei.
Ebner et al.	2001	Embryo fragmentation in vitro and its impact on treatment and pregnancy outcome	Retrospective	460 fresh embryo transfers.	Day-2 embryo transfers.	Mean patient age 32.5 +/- 4.9 years.	Long protocol combining GnRH with HMG. 5,000 - 10,000 IU of hCG was administered to induce ovulation.	Implantation and clinical pregnancy rate, obstetric and perinatal outcome.
Alikani et al.	1999	Human embryo fragmentation in vitro and its implications for pregnancy and implantation	Retrospective	2,410 patients.	Day-3 embryos.	Mean maternal age 35.7 +/- 4.25 years.	Stimulation with standard down-regulation protocol or a modification of the microflare protocol (Scott and Navot, 1994^2^) for pts ≥40 and those with previous response to down-regulation was unsatisfactory.	Degree and pattern of fragmentation on pregnancy and implantation.
Paternot et al.	2013	Semi-automated morphometric analysis of human embryos can reveal correlations between total embryo volume and clinical pregnancy	Prospective	458 patients; 2796 embryos.	Day-3 after IVF/ICSI.	All patients younger than 36 years.	Stimulation protocol as previously described by Debrock et al., 2010.^3^	Ongoing pregnancy (one intrauterine gestational sac at 20 weeks).

Racowsky et al., assessed if multiple evaluations of an embryo improve selection quality and thus implantation and pregnancy success. They noted that an increased level of fragmentation on both day-2 and -3 was associated with a significant reduction in the number of fetuses that developed to 12 weeks. They also noted that severe fragmentation (>50%) impaired overall embryo viability and may be related to low pregnancy rates and high risk of congenital malformations. The authors ultimately concluded that single day morphological evaluation on day-2 or day-3 has the same predictive value to a multi-day scoring system [22].

Another retrospective analysis of 460 fresh embryo transfers by Ebner et al., sought to determine the impact of embryo fragmentation on not just pregnancy, but also obstetric and perinatal outcomes. There was a significant relationship between fragmentation and implantation and clinical pregnancy rate, but not with multiple pregnancy rate or ongoing pregnancy rate [10]. Alikani et al., also studied embryo fragmentation and its implications for implantation and pregnancy rate and included fragmentation pattern into their discussion. They too found a significant decrease in implantation and pregnancy rate as the degree of fragmentation increased. They identified an effect on pregnancy rate when the degree of fragmentation was greater than 35%. The authors went on to discuss that not all fragmentations are detrimental to the embryo development and that the pattern of fragmentation matters. They found that fragmentation pattern type IV, defined as having large fragments distributed randomly and associated with uneven cells, had significantly lower implantation and clinical pregnancy rates when compared to types I-III. They concluded that detaching blastomere cytoplasm as large fragments is most detrimental to embryo development and implantation rate. In contrast, small, scattered fragments (type III) did not seem to appreciably affect the cell number or pose a serious threat to further development [7].

Lastly, Paternot et al., used sequential imaging techniques and a computer-assisted scoring system to study blastocyst development and the effect of fragmentation on clinical pregnancy. The authors reviewed the volume reduction over time as a measure of embryo fragmentation. They analyzed volumes on day-1 to -3 and found a significant association between total embryo volume and pregnancy rate on both day-2 (*P* = 0.003) and day-3 (*P* = 0.0003), with the total volume measured on day-3 being the best predictor of pregnancy outcome [92]. In contrast, Lahav-Baratz recently showed that there was no association between fragmentation rate and abortion or live birth rate. It was concluded that fragmented embryos still have implantation potential and could be considered for transfer when applicable [69].

### Effect of CF removal on embryo development

The effect of fragment removal on IVF outcomes has been controversial. Six of the studies included in this review discussed the impact of removing fragments on embryo development (Table 8) [7, 67, 93–96]. The literature is mixed, with some studies showing improvement in embryo development quality after fragmentation removal [7, 93], and others showing no difference at all [70, 94, 95].

**Table 8:** Effect of cytoplasmic fragmentation removal on embryo development

**Author**	**Year**	**Title of Paper**	**Type of study**	**No. of study participants**	**Embryo stage**	**Age**	**Intervention (stim protocol)**	**Outcome measure**
Alikani et al.	1999	Human embryo fragmentation in vitro and its implications for pregnancy and implantation	Retrospective	2,410 patients.	Day-3 embryos.	Mean maternal age 35.7 +/- 4.25 years.	Stimulation with standard down-regulation protocol or a modification of the microflare protocol (Scott and Navot, 1994^1^) for pts ≥40 and those with previous response to down-regulation was unsatisfactory.	Degree and pattern of fragmentation on pregnancy and implantation.
Eftekhari-Yazdi et al.	2006	Effect of fragment removal on blastocyst formation and quality of human embryos	Experimental	213 embryos.	4-6 cell surplus human embryos.	Age of female partners: Control (29.28 +/- 1.58 years), Experimental (28.98 +/- 1.71 years). Age of male partners: Control (35.22 +/- 3.66 years), Experimental (34.60 +/- 3.97 years)	Ovarian stimulation carried out following down-regulation as previously described by Porter et al., 1984.^2^	In-vitro development after blastomere fragmentation removal: day-6 size and number of blastomeres.
Keltz et al.	2006	Predictors of embryo fragmentation and outcome after fragment removal in in vitro fertilization	Retrospective	327 nondonor, fresh IVF cycles.	Assisted hatching and fragment removal on Day-3 embryos.	Cycles with fragmented embryos mean age 36.9 +/- 4.24 years. Cycles with no fragmented embryos mean age 35.4 +/- 4.74 years.	Controlled stimulation during IVF was performed using combination of GnRH agonists and gonadotropins in a long or short protocol for all subjects. Oocytes retrieved 35 hrs after 10,000 IU hCG was administered.	Predictors of fragmentation. Evaluated age, basal FSH and E(2) levels, the number of retrieved oocytes, and fertilization rates. Outcome assessments following defragmentation included rates of implantation, clinical pregnancy, spontaneous abortion, and live birth.
Keltz et al.	2010	Defragmentation of low grade day 3 embryos resulted in sustained reduction in fragmentation, but did not improve compaction or blastulation rates	Prospective	35 paired embryos (treatment and control group); 70 total embryos.	Defragmentation on Day-3 (6-8 cell stage); Fragmentation, compaction, morulation, and blastula formation evaluation on Day-4 or 5.		Controlled stimulation during IVF was performed using combination of GnRH agonists and gonadotropins in a long or short protocol for all subjects. Oocytes retrieved 35 hrs after 10,000 IU hCG was administered.	Day-5 embryo of fragmentation, compaction, morulation, blastulation formation rates.
Halvaei et al.	2016	Ultrastructure of cytoplasmic fragments in human cleavage stage embryos	Prospective	150 ICSI cycles.	Cleavage-stage embryos.	Female age: Control (29.1 +/- 3.7 years), Sham (31.4 +/- 4.9 years), Case (29.6 +/- 6 years). Male age: Control (33.64 +/- 5.3 years), Sham (35.56 +/- 6.7 years), Case (35 +/- 6.7 years).	Majority of patients underwent antagonist protocol for ovarian stimulation.	Evaluate ultrastructure of cytoplasmic fragment and perivitelline coarse granulation removal (cosmetic microsurgery) from embryos before embryo transfer on ART outcomes (rates of clinical pregnancy, live birth, miscarriage, multiple pregnancies, and congenital anomaly).
Yumoto et al.	2020	Removing the zona pellucida can decrease cytoplasmic fragmentations in human embryos: a pilot study using 3PN embryos and time-lapse cinematography	Retrospective	50 patients; 71 zygotes in which 3PN were confirmed after insemination.	ZP-free zygotes were cultured and observed continuously for 5 days in incubator equipped with time-lapse imaging system.	Mean maternal age 36.9 +/- 4.7 years.	?	Developmental morphology and embryonic quality.

Alikani et al., were one of the first investigators to define various patterns of fragmentation and perform microsurgical fragment removal to improve implantation potential [7]. The authors found that the pattern and degree of fragmentation, and not merely the presence of fragmentation, was significant. When assisted hatching and microsurgical fragment removal was performed, there was an overall 4% increase in implantation rate. They concluded that the removal of the fragments possibly restored the spatial relationship of the cells and limited the interference of cell-cell contact. Further, their preliminary data showed that blastocysts formed after fragment removal were better organized than their unmanipulated counterparts [7].

Eftekhari-Yazdi et al., similarly studied the effect of fragment removal on blastocyst formation and quality of embryos [93]. They compared day-2 embryos without removal of fragments to those that fragments were microsurgically removed. There were significantly higher quality embryos in defragmented group compared to the control. Furthermore, fragment removal improved the blastocyst quality compared to the control group. There was also a reduction of apoptotic and necrotic cells in experimental group when compared with the control group [93].

Two separate studies by Keltz et al., assessed implantation, clinical pregnancy, and birth outcomes after defragmentation [67], as well as embryo development and fragmentation rate after day-3 embryo defragmentation [94]. The authors first compared cycle outcomes between low-grade embryos that underwent micromanipulation for fragment removal (>10% fragmentation) and high-grade embryos that did not undergo defragmentation but were hatched on day 3. When compared, the defragmented group showed no difference in rates of implantation, clinical pregnancy, live birth, spontaneous abortion, or fetal defects as compared to the cycles that included all top-grade embryos. Factors associated with poor IVF prognosis and formation of embryo fragments included advanced age, decreased number of oocytes and embryos, and embryo grade [67].

A separate prospective randomized study by Keltz et al., looked more specifically at day-5 fragmentation, compaction, morulation and blastulation rates after low grade day-3 embryo defragmentation [94]. Paired embryos from the same patient, not intended to be transferred, were randomly placed in either the experimental group, assisted hatching and embryo defragmentation, or control group (assisted hatching alone). Paired embryos had no difference in mean cell number, percent fragmentation, and grade before randomization. Results showed that on day-5, embryos in the defragmentation group had significantly diminished fragmentation when compared with controls; however, there was no difference in compaction rate, morula formation rate or blastocyst formation rate. Embryo grade generally improved in the treatment group, but this was not statistically significant. Overall, in both groups, improved embryo development was significantly associated with lower levels of fragmentation in the day-3 embryos, supporting the idea that defragmented embryos maintain their reduced fragmented state throughout preimplantation development. Of note, this study had 35 embryos in each group and was limited to lower grade embryos not intended for transfer [94].

Another, larger prospective randomized study by Halvaei et al., compared the effect of microsurgical removal of fragments on ART outcomes. The authors divided 150 embryos with 10-50% fragmentation into three groups, case (*n*=50), sham (*n*=50), and control (*n*=50). They found no significant difference in rates of clinical pregnancy, miscarriage, live birth, multiple pregnancies, or congenital anomalies between these groups, ultimately showing that cosmetic microsurgery on preimplantation embryos to remove CFs had no beneficial effect [95].

Lastly, a pilot study by Yumoto et al., aimed to decrease CF in developing embryos by removing the zona pellucida of abnormally fertilized (3PN) donated oocytes [96]. Although they did not attempt to remove fragments themselves, this study is included as ZP-free oocytes are sometimes encountered in or because of ART procedures, i.e., ICSI. The results suggest that the rate of fragmentation is decreased after mechanical ZP removal. The authors concluded that ZP is not always necessary for normal embryo development since the ZP-free embryos developed normally, maintained their cell adhesions, and had a decreased rate of fragmentation [96]. It seems that defragmentation of an aneuploid or severely fragmented embryo, only improves the embryo morphology grade but the quality and fate of embryo is not changed [97].

### CF and chromosomal abnormalities in embryo

Although the relationship between DNA fragmentation and chromosomal abnormalities has been more commonly explored in the literature, CF may also be related to intrinsic chromosomal abnormalities in developing embryos. Fourteen studies included in this review explored this relationship (Table 9) [55, 56, 85, 98–108].

**Table 9:** Studies examining the relationship between cytoplasmic fragmentation and chromosomal abnormalities in human embryos

**Author**	**Year**	**Title of Paper**	**Type of study**	**No. of study participants**	**Embryo stage**	**PGT-A platform**	**Age**	**Intervention (stim protocol)**	**Outcome measure**
Findikli et al.	2004	Assessment of DNA fragmentation and aneuploidy on poor quality human embryos	Experimental	57 embryos.	Different developmental stages.	FISH	Female mean age: 30.4 +/- 5.9 years. Male mean age: 37.8 +/- 8.0 years.	Pituitary downregulation with GnRH analogue and follicular development stimulated with injection of FSH, HMG, and HCG.	Assess the extent of DNA fragmentation and aneuploidy in spare slow growing or arrested human embryos.
Munne et al.	1995	Embryo morphology, developmental rates and maternal age are correlated with chromosome abnormalities	Prospective	524 monospermic embryos; 283 analyzed with X, Y, 18, 13/21 probes and remainder with X, Y, and 18 probes.	Cleavage-stage embryos.	FISH	Maternal age groups used: 20 – 34, 35 – 39, 40 - 47 years.	Per protocol at Center for Reproductive Medicine and Infertility, The New York Hospital- Cornel University Medical College.	Detection of maternal age effect, complete assessment of mosaicism (by analysis of all cells), relationship with embryonic dysmorphism.
Almeida and Bolton	1996	The relationship between chromosomal abnormality in the human preimplantation embryo and development in vitro	Prospective	Total of 103 patients, 410 embryos.	Pronucleate to 8-cell stage.	Cytogenetic	Patient median age 33 years (range, 23-40 years).	Ovarian stimulation, ultrasound-directed follicle aspiration and IVF performed as described previously by Bolton et al., 1989.^1^	Preimplantation development rate of chromosomally-abnormal embryos. Association between chromosomal abnormalities and embryo morphology.
Magli et al.	2001	Chromosomal abnormalities in embryos	Retrospective	256 patients from S.I.S.M.E.R. Reproductive Medicine Unit; 1620 embryos biopsied.	Embryos at the 7-8 cell stage exhibited the lower frequency of abnormalities (55%) compared to embryos with a slower rate of cleavage (74%) at 3-4 cell stage.	FISH	Maternal age > 36 years.	PGD cycles following induction of multiple follicular growth as described by Ferraretti et al., 1996.^2^ Oocytes were transvaginally collected via ultrasound guidance 34-36 h after HCG administration.	Rate of development to expanded blastocysts.
Moayeri er al.	2008	Day-3 embryo morphology predicts euploidy among older subjects	Retrospective	1,043 biopsied blastomeres.	Day-3 embryos.	FISH	Mean age 37.7 years. AMA group mean age 40.1 +/- 2 years. Young group mean age 34.2 +/- 1 years.	Controlled ovarian hyperstimulation using a GnRH antagonist protocol with Follistim. Ovulation was triggered with 10,000 IU hCG given SC followed by transvaginal ultrasound–guided egg retrieval 34–36 hours later.	Day-3 embryo morphology score and PGD fluorescence in situ hybridization results for chromosomes: 13, 15, 16, 17, 18, 21, 22, X, and Y.
Baltaci et al.	2006	Relationship between embryo quality and aneuploidies	Retrospective	132 patients; 1107 embryos.	Day-3 embryos.	FISH	Mean age 35.45 +/- 5.6 years.	GnRH agonist given to assist flare-up effect. Tryptorelin acetate 0.1 mg subcutaneous daily or nafarelin acetate 200 µg intranasally 3x/day was administered for each patient. Stimulation with 300 IU follitropin alpha i.m. HCG 10,000 IU i.m. adminstered 36 h before oocyte retrieval.	Detect correlation between embryo quality and genetic status.
Pellestor et al.	1994	Relationship between morphology and chromosomal constitution in human preimplantation embryo	Prospective	462 preimplantation embryos analyzed (426 poor quality and 36 good quality embryos); chromosomal analysis successful in 153 cases.	Embryos examined 24 hrs and 48 hrs after insemination.	Cytogenetic			Chromosomal status and morphologic quality of preimplantation eggs.
Pellestor et al.	1994	Direct assessment of the rate of chromosomal abnormalities in grade IV human embryos produced by in-vitro fertilization procedure	Prospective	411 grade IV embryos from 327 couples participating in IVF.	Cleaved embryos classified 48 hrs after insemination.	Cytogenetic	Female age range 24 - 43 years. Male age range 25 - 52 years.	Stimulation protocol previously described by Audibert et al., 1989.^3^	Karyotype of grade IV embryos.
Chavez et al.	2012	Dynamic blastomere behavior reflects human embryo ploidy by the four-cell stage	Retrospective	85 two pronuclear and 25 cleavage-stage supernumerary human embryos.	Cleavage stage embryos; 4-cell stage.	aCGH	Average maternal age 33.5 years.		Karyotype of all blastomeres in 4-cell human embryos.
Ziebe et al.	2003	FISH analysis for chromosomes 13, 16, 18, 21, 22, X and Y in all blastomeres of IVF pre-embryos from 144 randomly selected donated human oocytes and impact on pre-embryo morphology	Retrospective	103 day 1 to day 3 embryos	Cleavage stage embryos	FISH,	Female age 25-37 years	Long or short protocols	Relationship between chromosomal status (13, 16, 21, 22, X and Y) and embryo morphology
Delimitreva et al.	2005	Chromosomal disorders and nuclear and cell destruction in cleaving human embryos	Retrospective	169 day 1 to day 3	Cleavage stage embryos	FISH	Mean maternal age: 33 years (range 23 - 38)	Long protocol	Relationship between chromosomal status (1, 5, 19 and X or 18, 21, X and Y) and blastomere fragmentation, nuclear apoptotic changes
Magli et al.	2007	Embryo morphology and development are dependent on the chromosomal complement	Retrospective	5227, 62 h after insemination	Cleavage stage embryos	FISH	maternal age ≥36 years	Long protocol	Relationship between embryo morphology and chromosomal competent
Vera-Rodriguez et al.	2015	Prediction model for aneuploidy in early human embryo development revealed by single-cell analysis	Retrospective	57	Day 3	aCGH	maternal age: 33.7±4.3 years	-	Using time-lapse, complete chromosomal assessment and single-cell RT–qPCR to simultaneously obtain a prediction model for aneuploidy
Minasi et al.	2016	Correlation between aneuploidy, standard morphology evaluation and morphokinetic development in 1730 biopsied blastocysts: a consecutive case series study	Case series study	1730	Day 5	aCGH	Maternal age: 36.8±4.24	Recombinant FSH and a GnRH agonist suppression protocol (short or long) or GnRH antagonist flexible protocol	Correlations among human blastocyst ploidy status, standard morphology evaluation and time-lapse kinetics

CF was rarely seen in embryos with normal chromosomal content. Findikli et al., studied DNA fragmentation and aneuploidy in poor quality embryos by TUNEL and fluorescent in situ hybridization (FISH) techniques. Within seven chromosomally abnormal embryos, each had variable degrees of CF [98]. This study suggests that DNA fragmentation, being a sign of chromosomal abnormalities, may exist together with CF.

An earlier study by Munne et al., examined 524 embryos using FISH analysis for three to five chromosomes. While controlling for age, they divided the embryos into three groups: arrested, slow and/or fragmented, or morphologically and developmentally normal. They found that polyploidy was the most common chromosomal abnormality in the arrested embryo group and decreased with increasing embryonic competence, with 44.5% polyploidy in arrested compared to 2.1% in morphologically normal embryos. Maternal age was not associated with polyploidy rates, but aneuploidy significantly increased with maternal age in morphologically normal human embryos [57]. Another early study by Almeida and Bolton also examined the relationship between chromosomal abnormalities and embryonic developmental potential. They found that cleavage-stage embryos with poor morphology, defined as irregular shaped blastomeres with severe fragmentation, showed a higher incidence of chromosomal abnormalities than those with good morphology [100]. Magli et al., found a more direct relationship between chromosomal abnormalities and embryo fragmentation in a larger retrospective study of nearly 1600 embryos. There was a strong association between percentage of fragmentation and chromosomal abnormalities (monosomies and trisomies), where 90% of chromosomal abnormalities were found in embryos with greater than 40% fragmentation [101].

Another retrospective study comparing maternal age to embryo morphology and chromosomal abnormalities was conducted by Moayeri et al., By examining nine chromosomes in day-3 embryos, they found that morphology predicted chromosomal status in the advanced maternal age group (≥38 years old), but not in younger patients. Fragmentation alone predicted euploidy in both the advanced maternal age and younger groups. This suggests that cellular fragmentation may be a predictor of chromosomal competence and thus embryo developmental potential [102]. 

In contrast, Baltaci et al., examined 1,000 embryos and concluded that embryo morphology was not predictive of euploidy and that a considerable number of chromosomally abnormal embryos with good development potential may be selected for embryo transfer. They used FISH for five chromosomes and found that a large proportion of both normal and aneuploid embryos were evaluated as top quality (grade I). For example, 66% of chromosomally abnormal embryos were of good quality (grade I and II). They found no significant difference among aneuploid embryos when distributed by age. However, a higher embryo quality found in normal compared to aneuploid embryos [103].

In addition, Pellestor et al., compared the relationship between morphology and chromosomal abnormalities in two separate studies. The first study found that aneuploidy was the most frequently observed abnormality after cytogenetic analysis of preimplantation embryos [55]. They defined the quality of embryos as good (grade I and II) and poor (grades III and IV). There was an increased chromosomal abnormality in poor quality embryos (84.3%) when compared to embryos with good quality (33.9%). Both aneuploidy and fragmentation were shown to be predominant in poor quality embryos, whereas mosaicism and polyploidy were the most frequent abnormalities in good quality embryos [55]. Pellestor et al., also performed cytogenetic analysis on 411 poor-quality embryos (grade IV) [85]. Ninety percent of the successfully analyzed cases showed abnormal chromosome complements, with aneuploidy being the most frequently observed. These results further support that a large majority of poor grade embryos are chromosomally abnormal and ultimately offer low chance of reproductive success for either embryo transfer or cryopreservation [85].

A separate study by Chavez et al., combined time-lapse imaging with karyotypic status of blastomeres in the 4-cell embryo to test whether blastomere behavior may reflect chromosomal abnormalities, using array comparative genomic hybridization (aCGH), during early cleavage [56]. In time-lapse observations, a large proportion of aneuploid and triploid, but not euploid embryos, exhibited cellular fragmentation. They showed that the probability of aneuploidy increased with higher fragmentation and only 65% of the fragmented embryo would be expected to form blastocyst. Furthermore, all the aneuploid embryos with additional unbalanced sub-chromosomal errors exhibited CF. The authors concluded that although fragmentation alone at a single point in time does not predict embryo developmental potential, time-lapse imaging with dynamic fragmentation screening may help detect embryonic aneuploidy [56].

Two more recent studies also used aCGH to evaluate the association between embryo ploidy and fragmentation. Vera-Rodriguez et al., in a retrospective study, compared the rate of embryo aneuploidy between two groups of high (≥25%) and low (˂25%) fragmentation. They found that the rate of aneuploidy in high and low fragmentation was 62.5 and 46.3%, respectively. However, the difference was not statistically significant concluding that using degree of fragmentation alone is not suggested to predict the embryo ploidy status [107]. Minasi et al., in a case series evaluated 1730 blastocyst ploidy with aCGH. They showed that there is no significant difference between day-3 embryo morphology and embryo ploidy. However, the quality of blastocyst (inner cell mass grade, trophectoderm grade, degree of expansion) was associated with embryo ploidy [106].

In a recent meta-analysis, it was shown there is trend between degree of fragmentation and rate of aneuploidy [109]. A major source of controversy in both early and recent studies on aneuploidy and fragmentation is the variation in the methods and criteria used to evaluate these factors. One of the aspects that differ across studies include the technique for detecting aneuploidy; FISH vs aCGH. Recent studies have used aCGH to detect aneuploidy and found no clear relationship in this regard. Also, the quality of the matching between groups, the design of the study (retrospective vs prospective), the timing of the fragmentation assessment, the use of time-lapse imaging to monitor the fate of fragments are the other reasons for this discrepancy. There is still the lack of a clear cut-off point for the percentage of fragmentation to predict aneuploidy. Further powerful studies using new methods like next gene sequencing and tile-lapse systems are recommended to shed light on the relationship between fragmentation and aneuploidy.

The literature highlights that poor quality embryos have a higher incidence of chromosomal abnormalities. Notably, CF is rarely observed in embryos with normal chromosomal content. Technological advancements, such as TLM, offer promising avenues to enhance our understanding and detection of embryonic aneuploidy. Overall, these studies underscore the complexity of the relationship between fragmentation and chromosomal abnormalities, emphasizing the need for continued research to refine embryo selection strategies and improve reproductive outcomes.

## Discussion and conclusion

The role of fragmentation in human embryo development and reproductive potential is widely recognized, albeit without standard definition nor agreed upon implication. While it has been shown that degree of fragmentation and embryo implantation potential are inversely proportional [5, 7, 9–21], the degree, pattern, and distribution of fragmentation as it relates to pregnancy outcome is debated in the literature. Our qualitative synthesis of 60 articles related to the study of embryo fragmentation and reproductive outcomes highlighted some of the challenges in analysis of fragmentation, while revealing trends in our evolving knowledge of how fragmentation may relate to functional development of the human embryo.

While fragmentation is best understood to be a natural process across species, the origin of fragmentation remains incompletely understood and likely multifactorial. Degree of fragmentation has been plausibly correlated to sperm DNA oxidation [37], errors in division [37], mitochondrial distribution [45], and overall embryo quality [39]. However, some causes of fragmentation are based on outdated studies and require validation in future research with higher quality and more advanced techniques. While cause of fragmentation remains a focus of investigation, advances in technology have allowed for more detailed analysis of its effect on embryo development and reproductive outcome. At the cellular level, increased fragmentation has been shown to be associated with higher rates of apoptosis, necrosis, and programmed cell death of cleavage-stage embryos [60–62]. Given the recognized significance of fragmentation on embryo development, it follows that many studies have been focused on IVF and ART impacts on fragmentation, as well as determining quantitative reproductive outcomes. In terms of other influences on degree of fragmentation, patient age was not universally found to be significantly associated with fragmentation [7, 70, 71] although age is certainly known to influence embryo quality. Most studies included in the search criteria showed that ART such as ICSI do not significantly impact fragmentation rate in developing embryos [74–76]. Those studies that found significant differences in embryo grading either between conventional fertilization and ICSI either did not find a difference in implantation or pregnancy rate or did not study it, suggesting that ICSI is not a significant contributor to poorer ART outcomes by way of embryo fragmentation.

In synthesizing the available data on ART and pregnancy outcomes with varying degrees of embryo fragmentation, most included studies did find a negative impact of increasing fragmentation on reproductive success while severe fragmentation does appear to be associated with poorer implantation rate and clinical pregnancy rate. This association may be related to the observation that increased fragmentation at the cleavage-stage embryo is related to chromosomal abnormalities incompatible with ongoing development or pregnancy.

The reviewed studies have several limitations. There are different grading systems in use that may impact detecting and reporting the degree of CF. Different criteria and terminology used in different studies may in turn make the comparison of outcome measures difficult. Another factor is the distribution pattern of CF. There are two types of scattered and concentrated fragments with different prognoses that is not considered in grading systems. Therefore, due to the lack of a standard cleavage-stage embryo grading system, comparing different studies should be done with caution. In addition, evaluation of embryo fragmentation is mostly based on individual observation which is subjective and has inter- and intra-observer subjectivity leading to high variable results even if performed by an experienced embryologist [110]. TLM is considered as a non-invasive tool and evaluates the embryo quality continuously and without the need to remove the embryo from the incubator [111]. The use of this technology allows for the analysis of embryo morphokinetics and has advanced knowledge of the developing embryo. Recently, artificial intelligence (AI) including machine learning and neural network has gained popularity in various fields of medicine including IVF and embryology. Accuracy of AI in prediction of fragmentation has been studied with encouraging results [112]. Further advances in technology will promote the use of AI as a tool in defining the effect of fragmentation on human embryo development and reproductive potential.

Although the precise origin and the importance of external or iatrogenic factors on fragmentation of cleavage-stage embryos varies in the literature, there is more consensus regarding severe fragmentation worsening reproductive outcomes. Given this important pattern, and the availability of increasingly sophisticated embryologic technology, further research is warranted to characterize more completely preventative or rescue techniques to improve reproductive outcomes.

## Data Availability

No datasets were generated or analysed during the current study.
